# Preparation and Evaluation of Orodispersible Tablets of Pheniramine Maleate by Effervescent Method

**DOI:** 10.4103/0250-474X.54284

**Published:** 2009

**Authors:** P. V. Swamy, S. P. Divate, S. B. Shirsand, P. Rajendra

**Affiliations:** Department of Pharmaceutics, H. K. E Society's College of Pharmacy, Sedam Road, Gulbarga-585 105, India

**Keywords:** Orodispersible tablets, pheniramine maleate, pregelatinized starch, sodium starch glycolate, croscarmellose sodium, crospovidone

## Abstract

In the present work, orodispersible tablets of pheniramine maleate were designed with a view to enhance patient compliance by effervescent method. In the effervescent method, mixture of sodium bicarbonate and tartaric acid (each of 12% w/w concentration) were used along with super disintegrants, i.e., pregelatinized starch, sodium starch glycolate, croscarmellose sodium and crospovidone. The prepared batches of tablets were evaluated for hardness, friability, drug content uniformity and *in vitro* dispersion time. Based on *in vitro* dispersion time (approximately 60 s), three formulations were tested for *in vitro* drug release pattern (in pH 6.8 phosphate buffer), short-term stability (at 40±2°/75±5% RH for 3 mo) and drug-excipient interaction (IR spectroscopy). Among three promising formulations, formulation ECP_4_ containing 4% w/w crospovidone and mixture of sodium bicarbonate and tartaric acid (each of 12% w/w) emerged as the overall best formulation (t_70%_ = 1.65 min) based on the *in vitro* drug release characteristics compared to commercial conventional tablet formulation. Short-term stability studies on the formulations indicated no significant changes in the drug content and *in vitro* dispersion time (P < 0.05).

Many patients express difficulty in swallowing tablets and hard gelatin capsules, tending to non-compliance and ineffective therapy[1]. Recent advances in novel drug devliery systems (NDDS) aim to enhance safety and efficacy of drug molecules by formulating a convenient dosage form for administration and to achieve better patient compliance. One such approach is orodispersible tablet[1–4]. Advantages of this drug delivery system include administration without water, accuracy of dosage, easy portability, alternative to liquid dosage forms, ideal for pediatric and geriatric patients and rapid onset of action. Pheniramine maleate is a member of alkylamine class of H_1_ receptor antagonists. It is an antihistamine used in the treatment of allergic conditions including urticaria and angioedema[5]. It was selected as drug candidate, as it is not available in such dosage form. Objective of the present study was to develop such a NDDS for pheniramine maleate by simple and cost- effective effervescent method.

Pheninamine maleate and super-disintegrants were gift samples from Aventis Pharma, Gujarat and Wockhardt Research Centre, Aurangabad, respectively. Directly compressible mannitol (Pearlitol SD 200) was a generous gift from Strides Areolabs, Bangalore, Microcrystalline cellulose (Alkem Labs, Mumbai), sodium bicarbonate (Ranbanxy Laboratories Limited), magnesium stearate and tartaric acid (S. D. Fine Chemicals, Mumbai) were used. All other chemicals used were of analytical reagent grade.

Orodispersible tablets of pheninamine maleate were prepared by effervescent method[6] according to the formulae given in Table 1. Sodium bicarbonate and tartaric acid were preheated at a temperature of 80° to remove absorbed/residual moisture and were thoroughly mixed in a mortar to get a uniform powder and then added to other ingredients. The ingredients after shifting through sieve No. 44 were thoroughly mixed in a tumbling cylindrical blender (fabricated in our laboratory). The blend thus obtained was directly compressed using 8 mm round flat beveled edge punches to get tablets of 150 mg weight on 10-station rotary tablet machine (Clit, Ahmedabad). A batch of 60 tablets was prepared for all the designed formulations.

**TABLE 1:** COMPOSITION OF DIFFERENT BATCHES OF ORODISPERSIBLE TABLETS OF PHENIRAMINE MALEATE

Ingredients (mg/tablet)	Formulation Code												
	
	Eo	EP_2_	EP_4_	EP_6_	EP_8_	ES_2_	ES_4_	ES_6_	ES_8_	ECC_2_	ECC_4_	ECP_2_	ECP_4_
Pheniramine maleate	12.5	12.5	12.5	12.5	12.5	12.5	12.5	12.5	12.5	12.5	12.5	12.5	12.5
Sodium bicarbonate	18	18	18	18	18	18	18	18	18	18	18	18	18
Tartaric acid	18	18	18	18	18	18	18	18	18	18	18	18	18
Pregelatinized starch	-	3	6	9	12	-	-	-	-	-	-	-	-
Sodium starch glycolate	-	-	-	-	-	3	6	9	12	-	-	-	-
Croscarmellose sodium	-	-	-	-	-	-	-	-	-	3	6	-	-
Crospovidone	-	-	-	-	-	-	-	-	-	-	-	3	6
Microcrystalline cellulose	30	30	30	30	30	30	30	30	30	30	30	30	30
Aspartame	6	6	6	6	6	6	6	6	6	6	6	6	6
Flavour (Pineapple)	3	3	3	3	3	3	3	3	3	3	3	3	3
Magnesium stearate	0.75	0.75	0.75	0.75	0.75	0.75	0.75	0.75	0.75	0.75	0.75	0.75	0.75
Purified talc IP	1.5	1.5	1.5	1.5	1.5	1.5	1.5	1.5	1.5	1.5	1.5	1.5	1.5
Pearlitol SD-200	60.25	57.25	54.25	51.25	48.25	57.25	54.25	51.25	48.25	57.25	54.25	57.25	54.25
Total Weight	150	150	150	150	150	150	150	150	150	150	150	150	150

Formulations EC_4_, ECC_2_ and ECP_4_ were selected as the best and used in further studies.

Twenty tablets were selected at random and weighted individually. The individual weights were compared with the average weight for determination of weight variation[7]. Hardness and friability of the tablets were determined by using a Monsanto Hardness Tester and a Roche Friabilator, respectively. For content uniformity test, ten tablets were weighed and powdered. The powder equivalent to 12.5 mg of pheniramine maleate was extracted into methanol and liquid was filtered. The pheninamine maleate content was determined by measuring the absorbance at 265 nm after appropriate dilution with methanol. The drug content was calculated using the standard calibration curve. The mean percent drug content was calculated as an average of three determinations[8]. For determination of *in vitro* dispersion time, one tablet was placed in a beaker containing 10 ml of pH 6.8 phosphate buffer at 37 ± 0.5° and the time required for complete dispersion was determined[9]. IR spectra of pheniramine maleate and its formulations were obtained by KBr pellet method using Perkin-Elmer FTIR series (model- 1615) spectrophotometer in order to rule out drug-carrier interactions.

*In vitro* dissolution of pheniramine maleate orodispersible tables was studied in USP XXIII type-II dissolution apparatus (Electrolab, Model- TDT 06N) employing a paddle stirrer at 50 rpm using 900 ml of pH 6.8 phosphate buffer at 37±0.5° as dissolution medium[10]. Aliquots of dissolution medium were withdrawn at specified intervals of time and analyzed for drug content by measuring the absorbance at 265 nm. The volume withdrawn at each time interval was replaced with fresh quantity of dissolution medium. Cumulative percent of pheniramine maleate released was calculated and plotted against time.

Short term stability studies on the promising formulation ECP_4_ were carried out by storing the tablets in an amber coloured rubber stoppered vial at 40±2°/75±5% RH over a 3 mo period. At intervals of one month, the tablets were visually examined for any physical changes, changes in drug content and *in vitro* dispersion time.

Orodispersible tablets of pheniramine maleate were prepared by effervescent method using pregelatinized starch (PGS), sodium starch glycolate (SSG), croscarmellose sodium (CC) and crospovidone (CP) as super-disintegrants while microcrystalline cellulose (MCC) and directly compressible mannitol (Pearlitol SD200) were used as diluent and sweetening agent respectively. The slight bitter taste of the drug has been masked by using 4% w/w of aspartame and 2% w/w of flavouring agent (spray dried pineapple flavour, Trusil). A total of 12 formulations and a control formulation E_o_ (without super-disintegrants) were designed.

As the material was free flowering (angle of repose values < 30° and Carr's index < 15), tablets obtained were of uniform weight (due to uniform die fill), with acceptable variation as per IP specifications i.e., below 7.5%. Drug content was found to be in the range of 97.52 to 101.56%, which is within acceptable limits. Hardness of the tablets was found to be 2.57 to 2.93 kg/cm^2^. Friability below 1.2% was an indication of good mechanical resistance of the tablets. Formulations ES_4_, ECC_2_ and ECP_4_ were found to be promising and displayed an *in vitro* dispersion time of approximately 40 s, which facilitates their faster dispersion in the mouth.

Among the tablet formulations employing various concentrations of PGS (2-8% w/w), SSG (2-8% w/w), CC (2-4% w/w) and CP (2-4% w/w) as super-disintegrants, the formulation ECP_4_ containing 4% w/w CP was found to be overall best promising formulation and has shown an *in vitro* dispersion time of 19 s, when compared to ES_4_, ECC_2_ and control (E_o_) formulations which show 35, 39 and 59 s values, respectively for the above parameter (Table 2). The performance of the above formulations based on *in vitro* dispersion time in decreasing order is as follows: ECP_4_ >ES_4_ >ECC_2_ >E_0_.

**TABLE 2:** EVALUATION OF ORODISPERSIBLE TABLETS

Formulations	Average weight* (mg) ± SD	Hardness* (kg/cm^2^)± SD	Friability (%)	Thickness* (mm)	Percent Drug content* ± SD	*In vitro* Dispersion time* (s) ± SD
E_0_	147±0.002	2.86±0.02	1.15	2.55±0.02	98.29±0.68	58.47±1.47
EP_2_	148±0.002	2.79±0.01	0.88	2.67±0.02	99.92±1.12	51.00±1.00
EP_4_	152±0.001	2.57±0.02	0.93	2.59±0.06	99.44±1.20	41.33±1.15
EP_6_	150±0.001	2.84±0.05	0.99	2.67±0.02	100.04±1.94	53.00±4.58
EP_8_	148±0.002	2.79±0.03	0.87	2.85±0.05	100.54±1.77	50.93±2.00
ES_2_	147±0.002	2.76±0.01	0.90	2.78±0.07	101.56±1.53	41.67±2.88
ES_4_	151±0.002	2.87±0.04	0.94	2.73±0.77	97.52±0.63	34.67±0.58
ES_6_	152±0.001	2.84±0.12	1.00	2.48±0.09	98.44±0.57	53.33±5.50
ES_8_	150±0.002	2.76±0.03	0.89	2.71±0.05	98.79±1.07	51.67±6.11
ECC_2_	149±0.001	2.78±0.04	0.90	2.83±0.02	100.18±0.01	38.67±2.51
Ecc_4_	148±0.002	2.93±0.01	0.98	2.75±0.04	99.85±1.99	39.87±0.23
ECP_2_	148±0.001	2.58±0.04	1.18	2.77±0.10	100.25±0.25	52.47±1.55
ECP_4_	151±0.002	2.83±0.02	0.95	2.58±0.03	98.18±0.16	18.91±1.39

* Average of three determinations. E_0_= Control formulation without super disintegrant, EP= Formulations containing pregelatinized starch as superdisintegrant, ES=Formulations containing sodium starch glycolate as superdisintegrant, ECC= Formulations containing croscarmellose sodium as superdisintegrant. ECP=Formulations containing crospovidone as superdisintegrant.

*In vitro* dissolution studies on the promising formulations (ES_4_, ECC_2_ and ECP_4_), the control (E_o_) and commercial formulation (CF) were carried out in pH 6.8 phosphate buffer, and the various dissolution parameter values viz., percent drug dissolved in 4 min (D_4_), dissolution efficiency at 4 min (DE_4min_)[11], t_50%_ and t_70%_ are shown in Table 3, and the dissolution profiles depicted in fig. 1. This data reveals that the formulations ES_4_ and ECP_4_ display almost similar results, and show 9 times greater dissolution efficiency and nearly 14 times faster drug release compared to the commercial formulation based on DE_4 min_ and t_70%_ values in pH 6.8 phosphate buffer. But as discussed earlier, upon considering the *in vitro* dispersion time also, ECP_4_ has been selected as the overall best formulation.

**TABLE 3:** *IN VITRO* DISSOLUTION PARAMETERS IN PH 6.8 PHOSPHATE BUFFER

Formulation Code	t_50%_ (min)	t_70%_ (min)	DE_4 min_ (%)	D_4_ (%)
E_o_	1.50	1.80	52.90	78.31
ES_4_	1.22	1.62	64.37	91.49
ECC_2_	1.25	1.75	58.00	79.54
ECP_4_	1.24	1.65	63.75	89.65
CF	13.75	22.25	7.03	15.05

CF= conventional commercial formulation, t_50%_ = time for 50% drug dissolution, t_70%_ = time for 70% drug dissolution, DE_4 min_ = dissolution efficiency in 4 min, D_4_= percent drug released in 4 min.

**Fig. 1:**
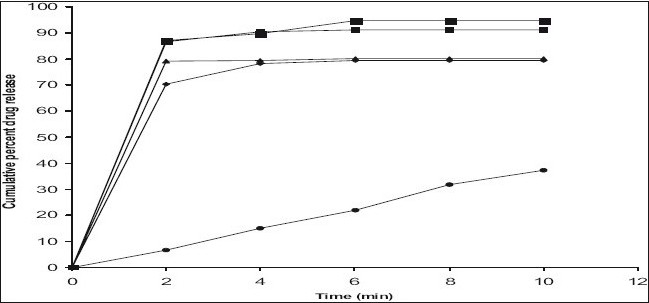
*In vitro* cumulative percent drug release vs time profiles *In vitro* cumulative percent drug release vs time profiles of promising pheniramine maleate formulations in pH 6.8 phosphate buffer. Plot showing cumulative percent drug release proflies of promising pheniramine maleate formulations, E0 (–◆–), ES4 (–■–), ECC2 (–▲–), ECP4 (–■–) and CF (–●–).

IR spectroscopic studies indicated that the drug is compatible with all the excipients. The IR spectrum of ECP_4_ showed all the characteristic peaks of pheniramine maleate pure drug, thus confirming that no interaction of drug occurred with the components of the formulation. Short-term stability studies of the above formulation indicated that there were no significant changes in drug content and *in vitro* dispersion time at the end of a 3 mo period (P < 0.05).
